# Supplementary material for: The Impact of Facility Surgical Caseload Volumes on Survival Outcomes in Patients Undergoing Radical Cystectomy
Source: Cancers (Basel). 2022 Dec 3;14(23):5984. doi: 10.3390/cancers14235984 (PMC9735798; doi:10.3390/cancers14235984)
Supplement: Supplementary file 1 [file cancers-14-05984-s001.zip › cancers-1987985-supplementary.pdf]

# Supplementary Material: The Impact of Facility Surgical Caseload Volumes on Survival Outcomes in Patients Undergoing Radical Cystectomy

**Giovanni E. Cacciamani [1,†], Afsaneh Barzi [2,†], Michael B. Eppler [1], Primo N. Lara Jr. [3], Chong-Xian Pan [4], Sumeet K. Bhanvadia [1], Parkash Gill [5], Monish Aron [1], Inderbir Gill [1] and Sarmad Sadeghi [6,*]**

1 Institute of Urology, USC Keck School of Medicine, Norris Comprehensive Cancer Center, University of Southern California, Los Angeles, CA 90033, USA
2 City of Hope Comprehensive Cancer Center, Department of Medical Oncology & Therapeutics Research, Duarte, CA 91010, USA
3 UC Davis Comprehensive Cancer Center, 4501 X Street, Sacramento, CA 95817, USA
4 Department of Medicine, Harvard Medical School, West Roxbury, MA 02132, USA
5 Department of Medicine, USC Norris Comprehensive Cancer Center, University of Southern California, Los Angeles, CA 90033, USA
6 Norris Cancer Hospital and Clinics Norris Comprehensive Cancer Center, University of Southern California, Los Angeles, CA 90033, USA
* Correspondence: sarmad.sadeghi@usc.edu; Tel.: +323-865-0553
† These authors contributed equally to this work.

**Table 3: Disease and treatment characteristics by volume group.**

** ni: neoadjuvant chemo indicated. ng: neoadjuvant chemo given. ai: adjuvant chemo indicated. ag: adjuvant chemo given. (0) no, (1) yes.*

*** These are patients who received neoadjuvant chemo without a clear indication, but post operatively based on pathology findings would have qualified for adjuvant chemo.*

| | FASC | | | | | FAC | | | | |
|---|---|---|---|---|---|---|---|---|---|---|
| | 1 | 2 | 3 | 4 | | 1 | 2 | 3 | 4 | |
| | <50th, [0-2] | 50-74th, [3-5] | 75-89th, [6-11] | 90th+, [12+] | p-value | <50th, [0-28] | 50-74th, [29-47] | 75-89th, [48-70] | 90th+, [71+] | p-value |
| **Radiation Surgery Sequence at any CoC Facility** | | | | | | | | | | |
| No radiation therapy and/or surgical procedures | 2655 (94.7%) | 4378 (95.5%) | 6151 (96.8%) | 13292 (98.2%) | <0.001 | 3631 (94.9%) | 4831 (96.4%) | 6422 (97.1%) | 11592 (98.1%) | <0.001 |
| Radiation therapy before surgery | 14 (0.5%) | 18 (0.4%) | 19 (0.3%) | 32 (0.2%) | | 19 (0.5%) | 18 (0.4%) | 16 (0.2%) | 30 (0.3%) | |
| Radiation therapy after surgery | 117 (4.2%) | 157 (3.4%) | 157 (2.5%) | 163 (1.2%) | | 151 (3.9%) | 136 (2.7%) | 142 (2.1%) | 165 (1.4%) | |
| Radiation therapy both before and after surgery | 0 (0.0%) | 1 (<1%) | 2 (<1%) | 4 (<1%) | | 1 (<1%) | 0 (0.0%) | 1 (<1%) | 5 (<1%) | |
| Intraoperative radiation therapy | 0 (0.0%) | 0 (0.0%) | 0 (0.0%) | 2 (<1%) | | 0 (0.0%) | 0 (0.0%) | 0 (0.0%) | 2 (<1%) | |
| Sequence unknown | 18 (0.6%) | 30 (0.7%) | 26 (0.4%) | 36 (0.3%) | | 26 (0.7%) | 26 (0.5%) | 30 (0.5%) | 28 (0.2%) | |
| **Surgical Margins Status at any CoC Facility- summarized** | | | | | | | | | | |
| Negative | 2219 (79.1%) | 3842 (83.8%) | 5428 (85.4%) | 11842 (87.5%) | <0.001 | 3171 (82.8%) | 4261 (85.0%) | 5552 (84.0%) | 10347 (87.5%) | <0.001 |
| Positive | 295 (10.5%) | 434 (9.5%) | 629 (9.9%) | 1155 (8.5%) | | 397 (10.4%) | 462 (9.2%) | 563 (8.5%) | 1091 (9.2%) | |
| Unknown | 290 (10.3%) | 308 (6.7%) | 298 (4.7%) | 532 (3.9%) | | 260 (6.8%) | 288 (5.7%) | 496 (7.5%) | 384 (3.2%) | |
| **Lymph Node Dissection Status** | | | | | | | | | | |
| 0 nodes examined | 548 (19.8%) | 672 (14.7%) | 714 (11.3%) | 1026 (7.6%) | <0.001 | 3171 (82.8%) | 4261 (85.0%) | 5552 (84.0%) | 10347 (87.5%) | <0.001 |
| <10 nodes examined | 1021 (36.9%) | 1905 (41.8%) | 2540 (40.1%) | 3285 (24.3%) | | 1437 (37.8%) | 1863 (37.4%) | 2228 (33.9%) | 3223 (27.3%) | |
| 10-14 nodes examined | 401 (14.5%) | 782 (17.1%) | 1179 (18.6%) | 2351 (17.4%) | | 646 (17.0%) | 831 (16.7%) | 1112 (16.9%) | 2124 (18.0%) | |
| 15-29 nodes examined | 523 (18.9%) | 837 (18.4%) | 1369 (21.6%) | 4667 (34.6%) | | 768 (20.2%) | 1129 (22.7%) | 1585 (24.1%) | 3914 (33.2%) | |
| 30+ nodes examined | 148 (5.3%) | 215 (4.7%) | 368 (5.8%) | 2037 (15.1%) | | 209 (5.5%) | 379 (7.6%) | 656 (10.0%) | 1524 (12.9%) | |
| Unknown | 127 (4.6%) | 150 (3.3%) | 160 (2.5%) | 136 (1.0%) | | 131 (3.4%) | 154 (3.1%) | 159 (2.4%) | 129 (1.1%) | |
| **Treatment Subgroup *** | | | | | | | | | | |
| ni(0) ng(0) ai(0) ag(0) | 590 (21.0%) | 1106 (24.1%) | 1567 (24.7%) | 3283 (24.3%) | <0.001 | 931 (24.3%) | 1217 (24.3%) | 1611 (24.4%) | 2787 (23.6%) | <0.001 |
| ni(0) ng(0) ai(0) ag(1) | 107 (3.8%) | 181 (3.9%) | 222 (3.5%) | 461 (3.4%) | | 124 (3.2%) | 175 (3.5%) | 294 (4.4%) | 378 (3.2%) | |
| ni(0) ng(0) ai(1) ag(0) | 259 (9.2%) | 424 (9.2%) | 659 (10.4%) | 1287 (9.5%) | | 383 (10.0%) | 466 (9.3%) | 643 (9.7%) | 1137 (9.6%) | |
| ni(0) ng(0) ai(1) ag(1) | 203 (7.2%) | 298 (6.5%) | 361 (5.7%) | 605 (4.5%) | | 254 (6.6%) | 320 (6.4%) | 383 (5.8%) | 510 (4.3%) | |
| ni(0) ng(1) ai(0) ag(0) | 49 (1.7%) | 81 (1.8%) | 136 (2.1%) | 416 (3.1%) | | 67 (1.8%) | 89 (1.8%) | 158 (2.4%) | 368 (3.1%) | |
| ni(1) ng(0) ai(0) ag(0) | 441 (15.7%) | 693 (15.1%) | 983 (15.5%) | 1851 (13.7%) | | 581 (15.2%) | 778 (15.5%) | 974 (14.7%) | 1635 (13.8%) | |
| ni(1) ng(0) ai(0) ag(1) | 126 (4.5%) | 167 (3.6%) | 233 (3.7%) | 445 (3.3%) | | 136 (3.6%) | 179 (3.6%) | 223 (3.4%) | 433 (3.7%) | |
| ni(1) ng(0) ai(1) ag(0) | 397 (14.2%) | 695 (15.2%) | 959 (15.1%) | 1976 (14.6%) | | 562 (14.7%) | 769 (15.3%) | 951 (14.4%) | 1745 (14.8%) | |
| ni(1) ng(0) ai(1) ag(1) | 333 (11.9%) | 461 (10.1%) | 638 (10.0%) | 1058 (7.8%) | | 417 (10.9%) | 517 (10.3%) | 608 (9.2%) | 948 (8.0%) | |
| ni(1) ng(1) ai(0) ag(0) | 142 (5.1%) | 199 (4.3%) | 260 (4.1%) | 888 (6.6%) | | 173 (4.5%) | 221 (4.4%) | 302 (4.6%) | 793 (6.7%) | |
| ni(1) ng(1) ai(1) ag(0) | 97 (3.5%) | 149 (3.3%) | 195 (3.1%) | 830 (6.1%) | | 120 (3.1%) | 160 (3.2%) | 264 (4.0%) | 727 (6.1%) | |
| ni(0) ng(1) ai(0/1) ag(0)- ret | 60 (2.1%) | 130 (2.8%) | 142 (2.2%) | 429 (3.2%) | | 80 (2.1%) | 120 (2.4%) | 200 (3.0%) | 361 (3.1%) | |
| **Multiagent chemotherapy as first course** | | | | | | | | | | |
| No chemo | 1476 (58.0%) | 2535 (61.4%) | 3642 (63.3%) | 7461 (60.8%) | <0.001 | 2162 (62.4%) | 2815 (62.3%) | 3661 (61.4%) | 6476 (60.2%) | <0.001 |
| Multiagent chemotherapy administered | 871 (34.2%) | 1299 (31.5%) | 1718 (29.9%) | 4090 (33.3%) | | 1047 (30.2%) | 1427 (31.6%) | 1887 (31.6%) | 3617 (33.6%) | |
| Single agent chemotherapy administered | 109 (4.3%) | 177 (4.3%) | 212 (3.7%) | 380 (3.1%) | | 150 (4.3%) | 162 (3.6%) | 215 (3.6%) | 351 (3.3%) | |
| Unknown number of agents administered | 90 (3.5%) | 115 (2.8%) | 181 (3.1%) | 349 (2.8%) | | 106 (3.1%) | 114 (2.5%) | 200 (3.4%) | 315 (2.9%) | |
| **90 Day Mortality 0: Patient alive at 90 days** | 2804 (100.0%) | 4584 (100.0%) | 6355 (100.0%) | 13529 (100.0%) | | 3828 (100.0%) | 5011 (100.0%) | 6611 (100.0%) | 11822 (100.0%) | |
| **No distant met at diagnosis** | 2804 (100.0%) | 4584 (100.0%) | 6355 (100.0%) | 13529 (100.0%) | | 3828 (100.0%) | 5011 (100.0%) | 6611 (100.0%) | 11822 (100.0%) | |
| **Urothelial carcinoma** | 2804 (100.0%) | 4584 (100.0%) | 6355 (100.0%) | 13529 (100.0%) | | 3828 (100.0%) | 5011 (100.0%) | 6611 (100.0%) | 11822 (100.0%) | |
| **Pathologic T2 or higher status** | 2106 (85.3%) | 3469 (83.2%) | 4703 (81.0%) | 9320 (73.5%) | <0.001 | 2859 (82.5%) | 3770 (82.4%) | 4694 (79.0%) | 8275 (74.2%) | <0.001 |
| **Clinical T2 or higher status** | 1495 (78.6%) | 2299 (76.6%) | 3193 (76.4%) | 6901 (71.6%) | <0.001 | 1938 (77.1%) | 2563 (77.3%) | 3239 (74.1%) | 6148 (72.2%) | <0.001 |
| **Pathologic T3 or higher status** | 1262 (51.1%) | 2000 (48.0%) | 2790 (48.0%) | 5723 (45.1%) | <0.001 | 1707 (49.3%) | 2190 (47.9%) | 2819 (47.4%) | 5059 (45.4%) | <0.001 |
| **Pathologic node+ status** | 622 (23.2%) | 964 (21.7%) | 1337 (21.5%) | 2932 (22.1%) | 0.36 | 860 (23.2%) | 1069 (22.0%) | 1373 (21.4%) | 2553 (22.0%) | 0.24 |
| **Clinical node+ status** | 164 (5.9%) | 245 (5.4%) | 295 (4.7%) | 627 (4.6%) | 0.016 | 233 (6.1%) | 252 (5.0%) | 319 (4.9%) | 527 (4.5%) | <0.001 |
| **N** | 2804 | 4584 | 6355 | 13529 | | 3828 | 5011 | 6611 | 11822 | |

**Figure S1.** Disease and Treatment Characteristics by Volume Group.

**Table S1.** Results by Facility Volume Groups.

| | Volume Groups | | | | | | | | | | |
|---|---|---|---|---|---|---|---|---|---|---|---|
| | FASC | | | | | | FAC | | | | |
| Variable | 1: <50th, [0-2] | 2: 50-74th, [3-5] | 3: 75-89th, [6-11] | 4: 90th+, [12+] | p-value | All | 1: <50th, [0-28] | 2: 50-74th, [29-47] | 3: 75-89th, [48-70] | 4: 90th+, [71+] | p-value |
| **N** | 2804 | 4584 | 6355 | 13529 | | 27,272 | 3828 | 5011 | 6611 | 11822 | |
| **Age groups** | | | | | | | | | | | |
| 1: <50 | 109 (3.9%) | 240 (5.2%) | 286 (4.5%) | 707 (5.2%) | 0.010 | 1,342 (4.9%) | 231 (6.0%) | 236 (4.7%) | 340 (5.1%) | 535 (4.5%) | 0.001 |
| 2: 50-64 | 889 (31.7%) | 1395 (30.4%) | 1971 (31.0%) | 4357 (32.2%) | | 8,612 (31.6%) | 1264 (33.0%) | 1582 (31.6%) | 2062 (31.2%) | 3704 (31.3%) | |
| 3: 65-74 | 1021 (36.4%) | 1673 (36.5%) | 2264 (35.6%) | 4711 (34.8%) | | 9,669 (35.5%) | 1332 (34.8%) | 1810 (36.1%) | 2365 (35.8%) | 4162 (35.2%) | |
| 4: 75+ | 785 (28.0%) | 1276 (27.8%) | 1834 (28.9%) | 3754 (27.7%) | | 7,649 (28.0%) | 1001 (26.1%) | 1383 (27.6%) | 1844 (27.9%) | 3421 (28.9%) | |
| **Sex** | | | | | | 0 (0.0%) | | | | | |
| 1: Male | 2102 (75.0%) | 3491 (76.2%) | 4863 (76.5%) | 10515 (77.7%) | 0.005 | 20,971 (76.9%) | 2887 (75.4%) | 3836 (76.6%) | 5041 (76.3%) | 9207 (77.9%) | 0.005 |
| 2: Female | 702 (25.0%) | 1093 (23.8%) | 1492 (23.5%) | 3014 (22.3%) | | 6,301 (23.1%) | 941 (24.6%) | 1175 (23.4%) | 1570 (23.7%) | 2615 (22.1%) | |
| **Race** | | | | | | | | | | | |
| White | 2570 (92.3%) | 4209 (92.3%) | 5815 (92.5%) | 12289 (92.1%) | 0.028 | 24,883 (91.2%) | 3436 (90.3%) | 4587 (92.4%) | 6092 (93.0%) | 10768 (92.4%) | <0.001 |
| Black | 156 (5.6%) | 259 (5.7%) | 366 (5.8%) | 727 (5.4%) | | 1,508 (5.5%) | 277 (7.3%) | 283 (5.7%) | 328 (5.0%) | 620 (5.3%) | |
| Other | 58 (2.1%) | 91 (2.0%) | 107 (1.7%) | 331 (2.5%) | | 587 (2.2%) | 90 (2.4%) | 95 (1.9%) | 131 (2.0%) | 271 (2.3%) | |
| **Primary Payor** | | | | | | | | | | | |
| 0: Not Insured | 90 (3.2%) | 188 (4.1%) | 223 (3.5%) | 299 (2.2%) | <0.001 | 800 (2.9%) | 201 (5.3%) | 180 (3.6%) | 173 (2.6%) | 246 (2.1%) | <0.001 |
| 1: Private Insurance | 869 (31.0%) | 1449 (31.6%) | 1989 (31.3%) | 4590 (33.9%) | | 8,897 (32.6%) | 1144 (29.9%) | 1570 (31.3%) | 2138 (32.3%) | 4045 (34.2%) | |
| 2: Medicaid | 141 (5.0%) | 208 (4.5%) | 271 (4.3%) | 524 (3.9%) | | 1,144 (4.2%) | 234 (6.1%) | 231 (4.6%) | 255 (3.9%) | 424 (3.6%) | |
| 3: Medicare | 1630 (58.1%) | 2657 (58.0%) | 3722 (58.6%) | 7633 (56.4%) | | 15,642 (57.4%) | 2129 (55.6%) | 2918 (58.2%) | 3729 (56.4%) | 6866 (58.1%) | |
| 4: Other Government | 37 (1.3%) | 32 (0.7%) | 55 (0.9%) | 147 (1.1%) | | 271 (1.0%) | 45 (1.2%) | 52 (1.0%) | 62 (0.9%) | 112 (0.9%) | |
| 9: Insurance Status Unknown | 37 (1.3%) | 50 (1.1%) | 95 (1.5%) | 336 (2.5%) | | 518 (1.9%) | 75 (2.0%) | 60 (1.2%) | 254 (3.8%) | 129 (1.1%) | |
| **Charlson-Deyo Score** | | | | | | | | | | | |
| 0 | 1973 (70.4%) | 3230 (70.5%) | 4316 (67.9%) | 9744 (72.0%) | <0.001 | 19,263 (70.6%) | 2698 (70.5%) | 3489 (69.6%) | 4699 (71.1%) | 8377 (70.9%) | 0.32 |

| | | | | | | | | | | | |
|---|---|---|---|---|---|---|---|---|---|---|---|
| 1 | 635 (22.6%) | 1040 (22.7%) | 1549 (24.4%) | 2922 (21.6%) | | 6,146 (22.5%) | 869 (22.7%) | 1154 (23.0%) | 1447 (21.9%) | 2676 (22.6%) | |
| 2 | 196 (7.0%) | 314 (6.8%) | 490 (7.7%) | 863 (6.4%) | | 1,863 (6.8%) | 261 (6.8%) | 368 (7.3%) | 465 (7.0%) | 769 (6.5%) | |
| **Facility Type** | | | | | | | | | | | |
| 1: Community Cancer Program | 984 (35.2%) | 586 (12.9%) | 142 (2.2%) | 15 (0.1%) | <0.001 | 1,727 (6.3%) | 1321 (34.8%) | 323 (6.5%) | 48 (0.7%) | 35 (0.3%) | <0.001 |
| 2: Comprehensive Community Cancer Program | 1506 (53.9%) | 2795 (61.3%) | 3303 (52.2%) | 1101 (8.2%) | | 8,705 (31.9%) | 1653 (43.5%) | 2950 (59.2%) | 2613 (39.7%) | 1489 (12.7%) | |
| 3: Academic/Research Program | 266 (9.5%) | 932 (20.5%) | 2175 (34.4%) | 10766 (80.1%) | | 14,139 (51.8%) | 794 (20.9%) | 1587 (31.9%) | 3441 (52.3%) | 8317 (70.8%) | |
| 4: Integrated Network Cancer Program | 38 (1.4%) | 244 (5.4%) | 702 (11.1%) | 1551 (11.5%) | | 2,535 (9.3%) | 33 (0.9%) | 119 (2.4%) | 475 (7.2%) | 1908 (16.2%) | |
| **Radiation Surgery Sequence at any CoC Facility** | | | | | | | | | | | |
| 0: No radiation therapy and/or surgical procedures | 2655 (94.7%) | 4378 (95.5%) | 6151 (96.8%) | 13292 (98.2%) | <0.001 | 26,476 (97.1%) | 3631 (94.9%) | 4831 (96.4%) | 6422 (97.1%) | 11592 (98.1%) | <0.001 |
| 2: Radiation therapy before surgery | 14 (0.5%) | 18 (0.4%) | 19 (0.3%) | 32 (0.2%) | | 83 (0.3%) | 19 (0.5%) | 18 (0.4%) | 16 (0.2%) | 30 (0.3%) | |
| 3: Radiation therapy after surgery | 117 (4.2%) | 157 (3.4%) | 157 (2.5%) | 163 (1.2%) | | 594 (2.2%) | 151 (3.9%) | 136 (2.7%) | 142 (2.1%) | 165 (1.4%) | |
| 4: Radiation therapy both before and after surgery | 0 (0.0%) | 1 (<1%) | 2 (<1%) | 4 (<1%) | | 7 (0.0%) | 1 (<1%) | 0 (0.0%) | 1 (<1%) | 5 (<1%) | |
| 5: Intraoperative radiation therapy | 0 (0.0%) | 0 (0.0%) | 0 (0.0%) | 2 (<1%) | | 2 (0.0%) | 0 (0.0%) | 0 (0.0%) | 0 (0.0%) | 2 (<1%) | |
| 9: Sequence unknown | 18 (0.6%) | 30 (0.7%) | 26 (0.4%) | 36 (0.3%) | | 110 (0.4%) | 26 (0.7%) | 26 (0.5%) | 30 (0.5%) | 28 (0.2%) | |
| **Surgical Margins Status at any CoC Facility-summarized** | | | | | | | | | | | |
| 0: Negative | 2219 (79.1%) | 3842 (83.8%) | 5428 (85.4%) | 11842 (87.5%) | <0.001 | 23,331 (85.5%) | 3171 (82.8%) | 4261 (85.0%) | 5552 (84.0%) | 10347 (87.5%) | <0.001 |
| 1: Positive | 295 (10.5%) | 434 (9.5%) | 629 (9.9%) | 1155 (8.5%) | | 2,513 (9.2%) | 397 (10.4%) | 462 (9.2%) | 563 (8.5%) | 1091 (9.2%) | |

| | | | | | | | | | | | |
|---|---|---|---|---|---|---|---|---|---|---|---|
| 2: Unknown | 290 (10.3%) | 308 (6.7%) | 298 (4.7%) | 532 (3.9%) | | 1,428 (5.2%) | 260 (6.8%) | 288 (5.7%) | 496 (7.5%) | 384 (3.2%) | |
| **Surgical Margins Status at any CoC Facility- detailed** | | | | | | | | | | | |
| 0: No residual tumor All margins are grossly and microscopically negative | 2219 (79.1%) | 3842 (83.8%) | 5428 (85.4%) | 11842 (87.5%) | <0.001 | 23,331 (85.5%) | 3171 (82.8%) | 4261 (85.0%) | 5552 (84.0%) | 10347 (87.5%) | <0.001 |
| 1: Residual tumor, NOS Involvement is indicated, but not otherwise specified | 136 (4.9%) | 165 (3.6%) | 212 (3.3%) | 388 (2.9%) | | 901 (3.3%) | 158 (4.1%) | 184 (3.7%) | 209 (3.2%) | 350 (3.0%) | |
| 2: Microscopic residual tumor Cannot be seen by the naked eye | 135 (4.8%) | 241 (5.3%) | 381 (6.0%) | 708 (5.2%) | | 1,465 (5.4%) | 211 (5.5%) | 247 (4.9%) | 328 (5.0%) | 679 (5.7%) | |
| 3: Macroscopic residual tumor, Gross tumor of the primary site which is visible to the naked eye | 24 (0.9%) | 28 (0.6%) | 36 (0.6%) | 59 (0.4%) | | 147 (0.5%) | 28 (0.7%) | 31 (0.6%) | 26 (0.4%) | 62 (0.5%) | |
| 7: Margins not evaluable, Cannot be assessed (indeterminate) | 123 (4.4%) | 146 (3.2%) | 142 (2.2%) | 210 (1.6%) | | 621 (2.3%) | 123 (3.2%) | 124 (2.5%) | 162 (2.5%) | 212 (1.8%) | |
| 9: Unknown or not applicable | 167 (6.0%) | 162 (3.5%) | 156 (2.5%) | 322 (2.4%) | | 807 (3.0%) | 137 (3.6%) | 164 (3.3%) | 334 (5.1%) | 172 (1.5%) | |
| **Lymph Node Dissection Status** | | | | | | | | | | | |
| 0: 0 nodes examined | 548 (19.8%) | 672 (14.7%) | 714 (11.3%) | 1026 (7.6%) | <0.001 | 23,331 (85.5%) | 3171 (82.8%) | 4261 (85.0%) | 5552 (84.0%) | 10347 (87.5%) | <0.001 |
| 1: <10 nodes examined | 1021 (36.9%) | 1905 (41.8%) | 2540 (40.1%) | 3285 (24.3%) | | 8,751 (32.1%) | 1437 (37.8%) | 1863 (37.4%) | 2228 (33.9%) | 3223 (27.3%) | |
| 2: 10-14 nodes examined | 401 (14.5%) | 782 (17.1%) | 1179 (18.6%) | 2351 (17.4%) | | 4,713 (17.3%) | 646 (17.0%) | 831 (16.7%) | 1112 (16.9%) | 2124 (18.0%) | |
| 3: 15-29 nodes examined | 523 (18.9%) | 837 (18.4%) | 1369 (21.6%) | 4667 (34.6%) | | 7,396 (27.1%) | 768 (20.2%) | 1129 (22.7%) | 1585 (24.1%) | 3914 (33.2%) | |
| 4: 30+ nodes examined | 148 (5.3%) | 215 (4.7%) | 368 (5.8%) | 2037 (15.1%) | | 2,768 (10.1%) | 209 (5.5%) | 379 (7.6%) | 656 (10.0%) | 1524 (12.9%) | |
| 5: Unknown | 127 (4.6%) | 150 (3.3%) | 160 (2.5%) | 136 (1.0%) | | 573 (2.1%) | 131 (3.4%) | 154 (3.1%) | 159 (2.4%) | 129 (1.1%) | |

| | | | | | | | | | | |
|---|---|---|---|---|---|---|---|---|---|---|
| **Treatment Cohort** | | | | | | | | | | |
| 0: ni(0) ng(0) ai(0) ag(0) | 590 (21.0%) | 1106 (24.1%) | 1567 (24.7%) | 3283 (24.3%) | <0.001 | 6,546 (24.0%) | 931 (24.3%) | 1217 (24.3%) | 1611 (24.4%) | 2787 (23.6%) | <0.001 |
| 1: ni(0) ng(0) ai(0) ag(1) | 107 (3.8%) | 181 (3.9%) | 222 (3.5%) | 461 (3.4%) | | 971 (3.6%) | 124 (3.2%) | 175 (3.5%) | 294 (4.4%) | 378 (3.2%) | |
| 2: ni(0) ng(0) ai(1) ag(0) | 259 (9.2%) | 424 (9.2%) | 659 (10.4%) | 1287 (9.5%) | | 2,629 (9.6%) | 383 (10.0%) | 466 (9.3%) | 643 (9.7%) | 1137 (9.6%) | |
| 3: ni(0) ng(0) ai(1) ag(1) | 203 (7.2%) | 298 (6.5%) | 361 (5.7%) | 605 (4.5%) | | 1,467 (5.4%) | 254 (6.6%) | 320 (6.4%) | 383 (5.8%) | 510 (4.3%) | |
| 4: ni(0) ng(1) ai(0) ag(0) | 49 (1.7%) | 81 (1.8%) | 136 (2.1%) | 416 (3.1%) | | 682 (2.5%) | 67 (1.8%) | 89 (1.8%) | 158 (2.4%) | 368 (3.1%) | |
| 8: ni(1) ng(0) ai(0) ag(0) | 441 (15.7%) | 693 (15.1%) | 983 (15.5%) | 1851 (13.7%) | | 3,968 (14.5%) | 581 (15.2%) | 778 (15.5%) | 974 (14.7%) | 1635 (13.8%) | |
| 9: ni(1) ng(0) ai(0) ag(1) | 126 (4.5%) | 167 (3.6%) | 233 (3.7%) | 445 (3.3%) | | 971 (3.6%) | 136 (3.6%) | 179 (3.6%) | 223 (3.4%) | 433 (3.7%) | |
| 10: ni(1) ng(0) ai(1) ag(0) | 397 (14.2%) | 695 (15.2%) | 959 (15.1%) | 1976 (14.6%) | | 4,027 (14.8%) | 562 (14.7%) | 769 (15.3%) | 951 (14.4%) | 1745 (14.8%) | |
| 11: ni(1) ng(0) ai(1) ag(1) | 333 (11.9%) | 461 (10.1%) | 638 (10.0%) | 1058 (7.8%) | | 2,490 (9.1%) | 417 (10.9%) | 517 (10.3%) | 608 (9.2%) | 948 (8.0%) | |
| 12: ni(1) ng(1) ai(0) ag(0) | 142 (5.1%) | 199 (4.3%) | 260 (4.1%) | 888 (6.6%) | | 1,489 (5.5%) | 173 (4.5%) | 221 (4.4%) | 302 (4.6%) | 793 (6.7%) | |
| 14: ni(1) ng(1) ai(1) ag(0) | 97 (3.5%) | 149 (3.3%) | 195 (3.1%) | 830 (6.1%) | | 1,271 (4.7%) | 120 (3.1%) | 160 (3.2%) | 264 (4.0%) | 727 (6.1%) | |
| 16: ni(0) ng(1) ai(0/1) ag(0)- retrospect | 60 (2.1%) | 130 (2.8%) | 142 (2.2%) | 429 (3.2%) | | 761 (2.8%) | 80 (2.1%) | 120 (2.4%) | 200 (3.0%) | 361 (3.1%) | |
| **Multiagent chemotherapy as first course** | | | | | | | | | | | |
| 0: No chemo | 1476 (58.0%) | 2535 (61.4%) | 3642 (63.3%) | 7461 (60.8%) | <0.001 | 15,114 (55.4%) | 2162 (62.4%) | 2815 (62.3%) | 3661 (61.4%) | 6476 (60.2%) | <0.001 |
| 1: Multiagent chemotherapy administered | 871 (34.2%) | 1299 (31.5%) | 1718 (29.9%) | 4090 (33.3%) | | 7,978 (29.3%) | 1047 (30.2%) | 1427 (31.6%) | 1887 (31.6%) | 3617 (33.6%) | |
| 2: Single agent chemotherapy administered | 109 (4.3%) | 177 (4.3%) | 212 (3.7%) | 380 (3.1%) | | 878 (3.2%) | 150 (4.3%) | 162 (3.6%) | 215 (3.6%) | 351 (3.3%) | |
| 3: Unknown number of agents administered | 90 (3.5%) | 115 (2.8%) | 181 (3.1%) | 349 (2.8%) | | 735 (2.7%) | 106 (3.1%) | 114 (2.5%) | 200 (3.4%) | 315 (2.9%) | |
| **90 Day Mortality 0: Patient alive, or died more than 90 days after surgery performed** | 2804 (100.0%) | 4584 (100.0%) | 6355 (100.0%) | 13529 (100.0%) | | 27,272 (100.0%) | 3828 (100.0%) | 5011 (100.0%) | 6611 (100.0%) | 11822 (100.0%) | |

| | | | | | | | | | | | |
|---|---|---|---|---|---|---|---|---|---|---|---|
| **No distant met at diagnosis** | 2804 (100.0%) | 4584 (100.0%) | 6355 (100.0%) | 13529 (100.0%) | | 27,272 (100.0%) | 3828 (100.0%) | 5011 (100.0%) | 6611 (100.0%) | 11822 (100.0%) | |
| **Urothelial carcinoma** | 2804 (100.0%) | 4584 (100.0%) | 6355 (100.0%) | 13529 (100.0%) | | 27,272 (100.0%) | 3828 (100.0%) | 5011 (100.0%) | 6611 (100.0%) | 11822 (100.0%) | |
| **Pathologic T2 or higher status** | 2106 (85.3%) | 3469 (83.2%) | 4703 (81.0%) | 9320 (73.5%) | <0.001 | 19,598 (71.9%) | 2859 (82.5%) | 3770 (82.4%) | 4694 (79.0%) | 8275 (74.2%) | <0.001 |
| **Clinical T2 or higher status** | 1495 (78.6%) | 2299 (76.6%) | 3193 (76.4%) | 6901 (71.6%) | <0.001 | 13,888 (50.9%) | 1938 (77.1%) | 2563 (77.3%) | 3239 (74.1%) | 6148 (72.2%) | <0.001 |
| **Pathologic T3 or higher status** | 1262 (51.1%) | 2000 (48.0%) | 2790 (48.0%) | 5723 (45.1%) | <0.001 | 11,775 (43.2%) | 1707 (49.3%) | 2190 (47.9%) | 2819 (47.4%) | 5059 (45.4%) | <0.001 |
| **Pathologic node status** | 622 (23.2%) | 964 (21.7%) | 1337 (21.5%) | 2932 (22.1%) | 0.36 | 5,855 (21.5%) | 860 (23.2%) | 1069 (22.0%) | 1373 (21.4%) | 2553 (22.0%) | 0.24 |
| **Clinical node status** | 164 (5.9%) | 245 (5.4%) | 295 (4.7%) | 627 (4.6%) | 0.016 | 1,331 (4.9%) | 233 (6.1%) | 252 (5.0%) | 319 (4.9%) | 527 (4.5%) | <0.001 |
